# Low Immunogenicity of Keratinocytes Derived from Human Embryonic Stem Cells

**DOI:** 10.3390/cells13171447

**Published:** 2024-08-29

**Authors:** Jiayi Shen, Xuanhao Zeng, Haozhen Lv, Yiting Jin, Yating Liu, Weiling Lian, Shiyi Huang, Qing Zang, Qi Zhang, Jinhua Xu

**Affiliations:** 1 Department of Dermatology, Huashan Hospital, Fudan University, Shanghai 200040, China; 2 The Shanghai Institute of Dermatology, Shanghai 200443, China; 3 Department of Dermatology, Beijing Hospital, National Center of Gerontology, Beijing 100730, China; 4 Institute of Geriatric Medicine, Chinese Academy of Medical Sciences, Beijing 100730, China; 5 Department of Thyroid and Breast Surgery, General Surgery, Huashan Hospital, Fudan University, Shanghai 200040, China

**Keywords:** keratinocytes, embryonic stem cells, differentiation, allograft rejection, immunogenicity

## Abstract

Epidermal transplantation is a common and widely used surgical technique in clinical medicine. Derivatives of embryonic stem cells have the potential to serve as a source of transplantable cells. However, allograft rejection is one of the main challenges. To investigate the immunogenicity of keratinocytes derived from human embryonic stem cells (ESKCs), we conducted a series of in vivo and in vitro experiments. The results showed that ESKCs have low HLA molecule expression, limited antigen presentation capabilities, and a weak ability to stimulate the proliferation and secretion of inflammatory factors in allogeneic PBMCs in vitro. In humanized immune mouse models, ESKCs elicited weak transplant rejection responses in the host. Overall, we found that ESKCs have low immunogenicity and may have potential applications in the field of regenerative medicine.

## 1. Introduction

Epidermal transplantation involves transplanting healthy skin tissue to the injured or deficient areas of the skin, such as in burn treatment, trauma repair, ulcer and post-surgery reconstruction following skin cancer removal, promoting wound healing, reducing the risk of infection, and improving the patient’s quality of life [1,2]. In 1869, Swiss surgeon Jacques-Louis Reverdin successfully performed the first autologous skin graft to treat a wound, which is considered the beginning of modern skin transplantation [3]. In 1981, Burke and Yannas et al. successfully applied artificial skin in clinical treatment for burns for the first time [4]. In recent years, tissue-engineered skin has been considered a highly promising skin substitute, capable of providing temporary protection for wounds and accelerating healing [5,6]. Additionally, 3D bioprinting technology plays a significant role in the precise fabrication of skin substitutes [7,8,9].

Rheinwald et al. successfully cultured keratinocytes in vitro, suggesting that small pieces of epithelium can be expanded into large amounts of cultured epithelium in vitro [10]. Building on this technique, our team developed a serum-free, feeder-free method for autologous skin culture and transplantation, which has already achieved excellent therapeutic results in the treatment of vitiligo [11]. Autologous epidermal transplantation typically requires using the patient’s own healthy skin tissue as the donor, but in some cases, like extensive burns or severe trauma, the patient’s skin may be insufficient for transplantation [12]. Human embryonic stem cells (hESCs) have the potential to differentiate into all cell types of the three embryonic germ layers [13]. Guenou et al. established a method for directing hESC differentiation into keratinocytes and reconstructing the full-thickness epidermis, and their findings indicate that keratinocytes differentiated from human embryonic stem cells may be a potential source of transplantable tissue [14].

The different cells derived from stem cells exhibit distinct immunogenic characteristics. For instance, cells such as pancreatic islet beta cells and retinal pigment epithelial cells derived from stem cells exhibit lower immunogenicity, while smooth muscle cells derived from stem cells show higher immunogenicity [15,16,17]. Human primary keratinocytes (PKCs) can elicit rejection responses in allogeneic transplantation, manifesting their immunogenicity [18,19]. So, it is crucial to elucidate the immunogenicity of keratinocytes derived from human embryonic stem cells (ESKCs), providing a fundamental basis for the clinical application of stem cell products.

In this study, we comprehensively assessed the immunogenicity of ESKCs through in vitro and in vivo experiments, examining aspects such as the cellular immune molecular expression levels and the capacity to stimulate T lymphocyte proliferation and secretion of inflammatory factors.

## 2. Methods

### 2.1. Cell Culture

The H9 human embryonic stem cell line used in this study was provided by the Stem Cell Bank, Shanghai Institutes for Biological Sciences, Chinese Academy of Sciences. Primary keratinocytes were extracted from normal human skin. This was conducted in accordance with ethical principles, and the details are provided at the end of the manuscript.

The embryonic stem cells were seeded onto T25 culture plates coated with Matrigel and cultured in mTeSR™ Plus medium in a 37 °C and 5% CO_2_ environment. The cell medium was changed every 2 days. Passages were conducted when the cell colonies reached 80% confluence. The primary keratinocytes were cultured in DKSFM in a 37 °C and 5% CO_2_ environment. The cell medium was changed every 2 days. Passages were conducted when the cell colonies reached 80% confluence.

Monocyte-derived dendritic cells (MoDCs) were induced to differentiate from the PBMCs obtained from healthy volunteer donors. This was conducted in accordance with ethical principles, and the details are provided at the end of the manuscript. The extracted PBMCs were seeded in 6-well plates at a density of 5 × 10^6^ cells/mL using monocyte adherence medium. After 2 h of adherence, the culture medium was aspirated, and the adherent monocytes were gently washed with warm monocyte adherence medium at 37 °C. Dendritic cell differentiation was initiated by adding RPMI 1640 medium containing 10% fetal bovine serum, human IL-4 (1 mg/mL), and GM-CSF (1 mg/mL). Half of the medium was changed every 3 days. On the sixth day, during the medium change, LPS (100 ng/mL) was added to induce cell maturation. The cells were harvested on the seventh day for subsequent analysis.

### 2.2. Differentiation from hESCs to Keratinocytes

A differentiation culture medium for the keratinocytes was prepared by adding 10 μM of retinoic acid (RA), 25 ng/mL of bone morphogenetic protein 4 (BMP4), 25 ng/mL of bone morphogenetic protein 7 (BMP7), 1× of Insulin-Transferrin-Selenium-Ethanolamine Supplement (ITS-X), 1μM of dexamethasone, and 10 ng/mL of insulin-like growth factor 1 (IGF-1) to DKSFM. The embryonic stem cells were cultured in this differentiation medium, with medium changes performed every 2 days. The first passage was performed when the cells covered the bottom of the plate, displaying characteristic dendritic structures, and specific markers for keratinocytes were detected by flow cytometry, typically around the 8th day of differentiation.

**Figure 1:**
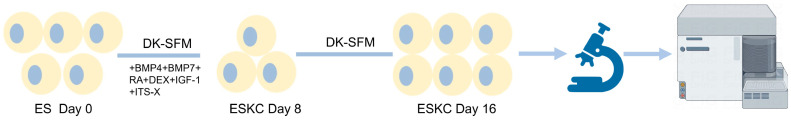
Diagram of the main differentiation process from hESCs to ESKCs. ESs, embryonic stem cells; ESKCs, keratinocytes derived from embryonic stem cells; DK-SFM, Defined Keratinocyte Serum Free Medium; BMP4, bone morphogenetic protein 4; BMP7, bone morphogenetic protein 7; RA, retinoic acid; DEX, dexamethasone; IGF-1, insulin-like growth factor 1; ITS-X, Insulin-Transferrin-Selenium-Ethanolamine Supplement.

After the cells were digested with Tryple, the cells were seeded into 6-well plates coated with type I collagen solution (30 ug/mL) at a density of 1 × 10^6^ cells per well using DKSFM. The cell medium was changed every 2 days. Passages were conducted when the cell colonies reached 80% confluence. The cells passaged to the second or third generation after differentiation were used for testing (Figure 1).

### 2.3. Stimulating the Expression of Immune Molecules

In order to stimulate potential immune responses and enhance the expression of immune molecules on cell surfaces, cultures of MoDC (positive control group), ESKC, and PKC were supplemented with 10 ng/mL of recombinant human IFN-γ and/or 25 ng/mL of TNF-α. Cellular analysis was conducted after 48 h of stimulation.

### 2.4. Immunofluorescence

Sterile poly-lysine slides in a 24-well plate held the cells. Once the desired density was reached, 4% paraformaldehyde fixed the cells for 10 min. Subsequently, the slides underwent blocking for 15 min with Quickblock™ immunostaining solution. After DPBS rinsing, the cells incubated with a diluted primary antibody in Phosphate-Buffered Saline with 0.1% Tween 20 (PBST) at room temperature for 1 h. Washed with PBST, the cells were exposed to secondary antibodies at room temperature for 1 more hour. Hoechst 33258 was added in the last 5 min of incubation. Lastly, the cells were washed and viewed under a fluorescence confocal microscope (Supplement Table S1).

### 2.5. Flow Cytometry

The cells underwent a DPBS wash, a 30 min antibody incubation, and another DPBS wash. For intracellular staining, the cells were fixed with 0.01% paraformaldehyde for 10 min, followed by a 15 min PBS treatment for membrane permeabilization. Subsequently, in darkness at 4 °C, the cells were incubated with primary antibodies diluted in PBS for 20 min. After multiple PBS washes, the cells received fluorescent secondary antibodies in PBS. After a 20 min dark incubation, cells were washed and analyzed via BD LSRFortessa flow cytometry. The data analysis employed Flowjo v10.4. The panel for flow cytometry is referenced in the Supplement (Supplement Figure S1). For each experiment, three independent experiments were performed.

### 2.6. qPCR Analysis

Total RNA extraction was initiated using the RNAeasy™ Animal RNA Isolation Kit (QIAGEN, Hilden, Germany) according to the instructions. RNA concentration was quantified through the Biotek Synergy H1 system. The absorbance of a diluted RNA sample was measured at 260 and 280 nm. Reverse transcription was performed using the Fast All-in-One RT Kit (ABM, Richmond, BC, Canada). After treating the RNA samples with DNase, the reverse transcription reaction system was set up according to the instructions. The reaction was carried out at 42 °C for 15 min. Subsequently, the sample cDNA was obtained after dilution. The primer sequences were downloaded from PrimerBank (https://pga.mgh.harvard.edu/primerbank/ (accessed on 27 November 2022)), and primer synthesis was purchased from the Genewiz company (South Plainfield, NJ, USA) (Supplement Table S2). Subsequently, qPCR experiments were executed on Quantstudio 7 Flex with the 2x Super SYBR Green qPCR Master Mix. The program was set as follows: 95 °C for 5 s, 60 °C for 34 s, repeated 40 times, followed by 95 °C for 15 s, 60 °C for 15 s, and finally 4 °C. The data were analyzed employing the 2^−ΔΔct^ method, utilizing GAPDH and TBP as reference genes for keratinocytes and PBMC, respectively. For each experiment, three independent experiments were performed.

### 2.7. Mixed Lymphocytes Reaction

The mixed lymphocytes reaction experiment was divided into five groups, with PBMCs from healthy volunteer A used as the responder cells in each group. Groups were categorized based on the type of stimulating cells (Table 1). Cell proliferation was assessed using CellTrace Violet dye (C34557, Thermofisher, Waltham, MA, USA). Cells from each group were separately added to a 96-well plate, with each well containing 1 × 10^5^ stimulating cells and 1 × 10^6^ responder cells. The cells were co-cultured for 72 h in complete RPMI 1640 medium containing 10% serum. After co-culture, each group of cells underwent assessments through flow cytometry and qPCR. The supernatant from each group was subjected to protein chip analysis. Each experiment was replicated three times.

### 2.8. Protein Microarray

An amount of 1 mL of supernatant was collected from each group in the co-culture system and analyzed using the Human Inflammation Array (QAH-INF-1) from Ray Biotech, Peachtree Corners, GA, USA. The specific methods are as follows, according to the instructions: After digestion with lysis solution, samples were centrifuged at 4 °C. The post-lysis protein concentration was assessed via BCA assay. The quantitative antibody chip, sealed with dilution buffer, received a 100 µL sample per well for overnight incubation. After incubation, washing ensued using buffer I and the chip washing station. The antibody mix was diluted with dilution buffer, added to the detection wells, and incubated at room temperature for 2 h. Subsequently, washing occurred, and diluted Cy3-streptavidin was added, and it was incubated in subdued light for 1 h. Cy3 channel fluorescence was detected using an InnoScan 300 microarray scanner (Innopsys, Carbonne, France), with data recorded. For each experiment, three independent experiments were performed.

### 2.9. Transcriptome Sequencing

Transcriptome sequencing was conducted on three samples of ESKC. The specific methods are as follows: Transcriptome libraries were constructed, and the RNA integrity was assessed using the Agilent 2100 Bioanalyzer (Agilent Technologies, Santa Clara, CA, USA). The purification of the products was performed using AMPure XP magnetic beads (Beckman, Indianapolis, IN, USA), and library quantification was carried out using a fluorometer. The libraries were diluted to a concentration of 1.5 ng/μL, and insert sizes were assessed using the Agilent 2100 Bioanalyzer (Agilent Technologies, Santa Clara, CA, USA). Following library quality checks, different libraries were separated and subjected to 150 bp sequencing using the Illumina NovaSeq 6000 platform (Illumina, San Diego, CA, USA). After data quality control, further computational analysis was performed on R software (v4.1.2). ESKC data were uploaded to the Gene Expression Omnibus public database. The PKC sequencing data from GSE216519 was used as control. Differential gene expression between ESKCs and PKCs was determined using limma (v3.50.0), with a threshold set at adjusted *p*-value < 0.05 for identifying differentially expressed genes (DEGs) [20]. The GO (Geneontology) functional enrichment analysis was conducted using the Hiplot online platform (https://hiplot.com.cn/ (accessed on 30 November 2022)). For each experiment, three independent experiments were performed.

### 2.10. Humanized Immune Mouse Model Experiment

Female NOD.Cg-prkdc^scid^iL2rg^tm1sug^/JicCrl (MHC-I/MHC-II knock out) immunodeficient mice at 6 weeks of age were purchased from Charles River Co. (Beijing, China), and the strain was introduced by the Central Institute for Experimental Animals (CIEA) in Japan and has been officially authorized. On day 0, human PBMCs were intravenously injected via the tail vein at a dose of 5 × 10^6^ cells per mouse. The mice were housed in a specific pathogen-free (SPF) environment. Model identification was conducted 3–4 weeks after the injection of human PBMCs. Peripheral blood from the mice was collected for flow cytometry analysis to detect the expression of human CD45 (hCD45) and mouse CD45 (mCD45) (Supplement Figure S2). The percentage of hCD45 was calculated, and successful modeling was indicated if hCD45 constituted 30% or more of the total hCD45+mCD45 count. The successfully modeled mice were randomly divided into two groups, with each group receiving injections of either ESKCs or PKCs (Supplement Figure S2). Each group consisted of 3 mice. Two types of keratinocytes were separately cultured, digested, and counted during the logarithmic growth phase. The cells were then resuspended in PBS buffer at a concentration of 1 × 10^6^ cells/100 μL. An amount of 100 μL of cell suspension was injected subcutaneously into the right dorsal area of each humanized immune mouse using a 1-milliliter syringe in the SPF environment. An equal volume of cell-free PBS was injected on the opposite site, and the injection sites were marked with a marker pen. One week after the transplantation of keratinocytes, skin samples from the grafting site and the opposite site were surgically removed and fixed in 4% paraformaldehyde. Both HE staining and immunofluorescence staining were performed according to standard protocol. The spleens were harvested from the mice, and single-cell suspensions were obtained by grinding. After red blood cell lysis and washing with PBS, further tests were conducted. All animal experiments comply with the regulations of the Ethics Committee of Fudan University.

### 2.11. Statistical Analysis

The significance level of inter-sample comparisons was tested using an ANOVA (n ≥ 3) or *t*-test (n = 2). Differences were considered statistically significant when *p* < 0.05. The smaller the *p*-value, the stronger the significance. We distinguish this in the figures using asterisks: * for *p* < 0.05; ** for *p* < 0.01; *** for *p* < 0.001; **** for *p* < 0.0001; and ns for no statistical significance. All data are presented as mean ± standard deviation. Data analysis was performed using Graphpad Prism v6.0 software.

## 3. Results

### 3.1. Identification of hESC-Derived Keratinocytes

**Figure 2:**
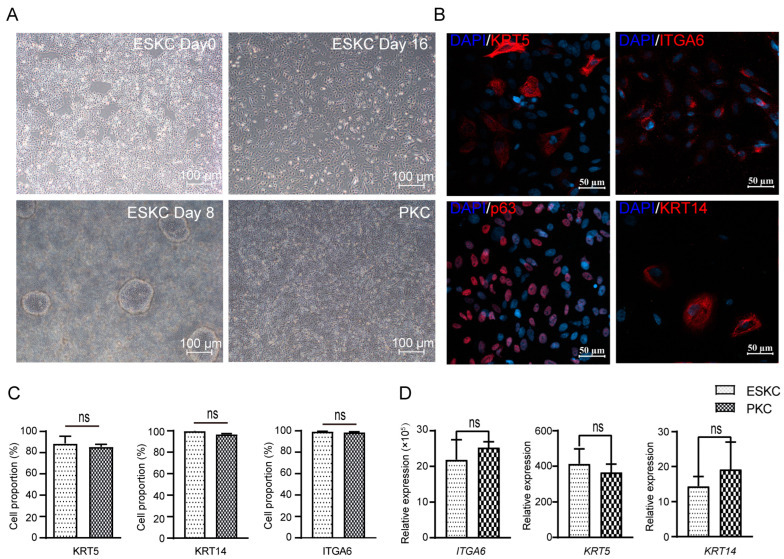
The differentiation from hESCs to ESKCs and the characterization of ESKCs. (**A**) Morphological changes of hESC in differentiation under inverted phase contrast microscope. (**B**) Expression of critical keratinocyte-associated proteins under fluorescence confocal microscope. (**C**) Expression of key keratinocyte-associated markers were detected by flowcytometry. (**D**) Gene transcriptions of key keratinocyte-associated markers were detected by qPCR. The hESCs were set as a reference sample, and GAPDH was set as the reference gene. Data in the figures are presented as mean ± S.D of n = 3; ns, no statistical significance.

hESCs underwent directed differentiation into keratinocytes through the induction of certain cytokines. The cells derived from hESCs grew as oval or polygonal shapes characterized by distinct boarders, resembling PKCs (Figure 2A). The differentiated cells showed high expression levels of KRT5, p63, and ITGA6, characteristic protein markers of keratinocytes in immunofluorescence staining (Figure 2B). Flow cytometry revealed that differentiated cells exhibited high expression levels of ITGA6, KRT5, and KRT14, and cells with high expression of these markers accounted for 80–95% of the total cell population (Figure 2C,D). These findings confirmed the successful induction of directed differentiation from hESCs to keratinocytes.

### 3.2. Low Gene Expression of ESKCs in Antigen Presentation and Interferon Response Pathway

**Figure 3:**
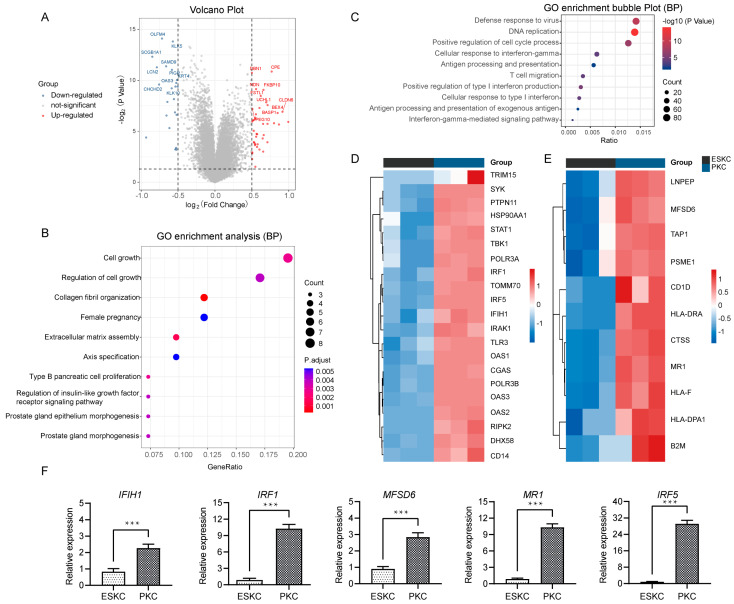
Transcriptomic differential gene pathway analysis and validation between ESKCs and PKCs. (**A**) A volcano plot showing the DEGs in ESKCs compared to PKCs. The dots above the horizontal line represent DEGs (adjusted.*p*.value < 0.05), with the red color representing pronounced upregulated genes and blue indicating pronounced downregulated genes. (**B**) A bubble plot for GO functional enrichment analysis of upregulated genes in ESKCs compared to PKCs, with the bubble color representing significance and the bubble size representing the number of genes enriched in that pathway. (**C**) A bubble plot for GO functional enrichment analysis of downregulated genes in ESKCs compared to PKCs, with the bubble color representing significance and the bubble size representing the number of genes enriched in that pathway. (**D**) A heatmap comparing the gene expression levels related to the cellular response to the interferon-gamma pathway, whose cell colors indicate gene expression levels, with red indicating upregulated genes (darker red indicating higher expression) and blue indicating downregulated genes (darker blue indicating lower expression). (**E**) A heatmap comparing the gene expression levels related to the antigen processing and presentation of the exogenous antigen pathway, whose cell colors indicate gene expression levels, with red indicating upregulated genes (darker red indicating higher expression) and blue indicating downregulated genes (darker blue indicating lower expression). (**F**) The validation of key molecules in the above pathways by qPCR. The relative expression levels indicate the mRNA relative expression levels of each group relative to the reference gene GAPDH within the ESKC group. Between-group comparisons were conducted using two-way ANOVA and Tukey’s post hoc multiple comparison statistical tests. Data in the figures are presented as mean ± S.D of n = 3; ***, *p* < 0.001.

Next, we compared the qualified sequencing data of ESKCs with the downloaded PKC sequencing data. The differential analysis showed that 3389 genes were significantly upregulated, while 3293 were significantly downregulated (Figure 3A). GO analysis revealed that the upregulated genes were involved in regulation of cell growth, collagen fibril organization, extracellular matrix assembly (Figure 3B). Notably, the major downregulated genes in ESKCs were enriched in pathways related to antiviral immune response, cellular response to interferon-gamma, antigen processing and presentation, and positive regulation of type I interferon production (Figure 3C). IFN-γ is a prototypical proinflammatory cytokine that plays a central role in inflammation and acute graft rejection. Specifically, in the cellular response to interferon pathway, ESKCs exhibited downregulation of genes such as IRF1, IRF5, IFIH1, relative to PKCs (Figure 3D). In the antigen processing and presentation pathway, ESKCs showed downregulation of genes including MFSD6 and MR1 (Figure 3E). The qPCR results also demonstrated significant downregulation of IFIH1, IRF1, IRF5, MFSD6, and MR1 gene expression levels in ESKCs relative to PKCs (Figure 3F).

### 3.3. ESKCs Showed Low Expression of HLA Molecules and Costimulatory Molecules

Dendritic cells are professional antigen-presenting cells characterized by high expression of HLA molecules and co-stimulatory molecules on their surface. Dendritic cells derived from mononuclear cells (MoDC) were used as positive control [21].

**Figure 4:**
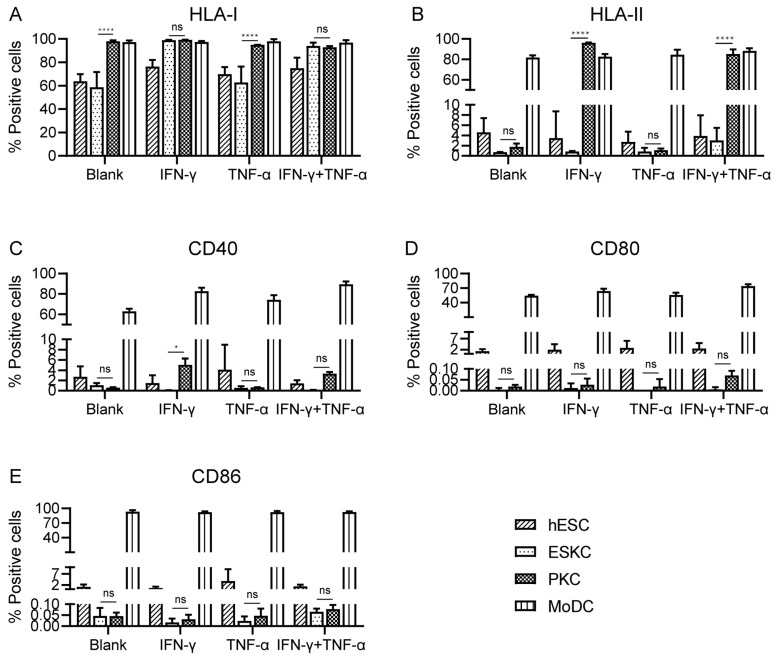
The comparison of the expression of immune-stimulating molecules in various cell types. (**A**) Expression of HLA-I molecules on hESCs, ESKCs, PKCs, and MODCs after different stimulation. (**B**) Expression of HLA-II molecules on hESCs, ESKCs, PKCs, and MODCs after different stimulation. (**C**) Expression of CD40 on hESCs, ESKCs, PKCs, and MODCs after different stimulation. (**D**) Expression of CD86 on hESCs, ESKCs, PKCs, and MODCs after different stimulation. (**E**) Expression of CD80 on hESCs, ESKCs, PKCs, and MODCs after different stimulation. MoDC, mononuclear cell-derived dendritic cells. A two-way ANOVA followed by Tukey’s multiple comparison test was performed to make intergroup comparisons. Data in the figures are presented as mean ± S.D of n = 3. *, *p* < 0.05; ****, *p* < 0.0001; ns, no statistical significance.

With no inflammatory cytokine stimulation, PKCs showed high expression levels of HLA-I molecules, and the expression levels of HLA-I molecules in ESKCs were significantly lower than those of PKCs. After TNF-α stimulation, there was no significant change in HLA-I expression. Upon IFN-γ stimulation, HLA-I molecules on ESKCs were upregulated, and PKCs maintained high expression levels of HLA-I molecules (Figure 4A, Supplement Figure S3).

With no inflammatory cytokine stimulation, both ESKCs and PKCs exhibited low expression of HLA-II molecules. After TNF-α stimulation, ESKCs and PKCs maintained low expression of HLA-II molecules. Interestingly, under IFN-γ stimulation, PKCs expressed a significantly high level of HLA-II molecules, while ESKCs still displayed low expression of HLA-II molecules (Figure 4B, Supplement Figure S3).

With no inflammatory cytokine stimulation, both ESKCs and PKCs exhibited low expression of co-stimulatory molecules CD40, CD80, and CD86. After stimulation with TNF-α or IFN-γ, there was no significant change in the expression of co-stimulatory molecules CD40, CD80, and CD86 in ESKCs and PKCs (Figure 4C–E).

### 3.4. ESKCs Exhibited a Limited Capacity to Stimulate Proliferation of T Lymphocytes and Secretion of Inflammatory Factors In Vitro

**Figure 5:**
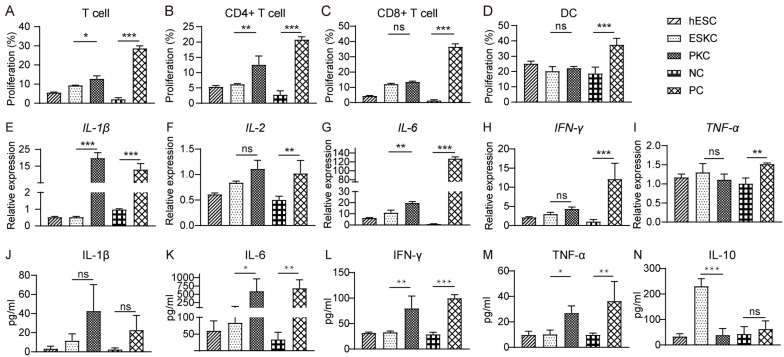
In vitro comparison of immunogenicity between ESKCs and PKCs. (**A**) The proliferation of T lymphocytes in the mixed lymphocytes reaction system with ESKCs or PKCs. (**B**) The proliferation of CD4^+^ T lymphocytes in the mixed lymphocytes reaction system with ESKCs or PKCs. (**C**) The proliferation of CD8^+^ T lymphocytes in the mixed lymphocytes reaction system with ESKCs or PKCs. (**D**) The proliferation of dendritic cells in the mixed lymphocytes reaction system with ESKCs or PKCs. (**E**) Comparison of mRNA transcription of IL-1β produced by the immune cells in the mixed lymphocytes reaction system. (**F**) Comparison of mRNA transcription of IL-2 produced by the immune cells in the mixed lymphocytes reaction system. (**G**) Comparison of mRNA transcription of IL-6 produced by the immune cells in the mixed lymphocytes reaction system. (**H**) Comparison of mRNA transcription of IFN-γ produced by the immune cells in the mixed lymphocytes reaction system. (**I**) Comparison of mRNA transcription of TNF-α produced by the immune cells in the mixed lymphocytes reaction system. (**J**) Comparison of protein levels of IL-1β produced by the immune cells in the mixed lymphocytes reaction system. (**K**) Comparison of protein levels of IL-6 produced by the immune cells in the mixed lymphocytes reaction system. (**L**) Comparison of protein levels of IFN-γ produced by the immune cells in the mixed lymphocytes reaction system. (**M**) Comparison of protein levels of TNF-α produced by the immune cells in the mixed lymphocytes reaction system. (**N**) Comparison of protein levels of IL-10 produced by the immune cells in the mixed lymphocytes reaction system. Inter-group comparisons were conducted using a two-way ANOVA and Tukey’s post hoc multiple comparison statistical tests. Data in the figures are presented as mean ± S.D of n = 3; *, *p* < 0.05; **, *p* < 0.01, ***, *p* < 0.001; ns, no statistical significance.

In mixed lymphocytes reaction experiments, stimulation of ESKCs resulted in weaker proliferation of T lymphocytes compared to that in PKCs (Figure 5A), which was mainly characterized by lower proliferation of CD4^+^ T lymphocytes but not CD8^+^ T lymphocytes (Figure 5B,C). Dendritic cell proliferation showed no significant difference between the ESKC and PKC groups (Figure 5D). After ESKC stimulation, the gene expression levels of IL-1β and IL-6 were significantly lower compared to PKC stimulation (Figure 5E,G). The secretion levels of IL-6, TNF-α, and IFN-γ in the ESKC group were significantly lower than those in the PKC group (Figure 5K–M).

### 3.5. Low Immune Responsiveness of ESKCs In Vivo

**Figure 6:**
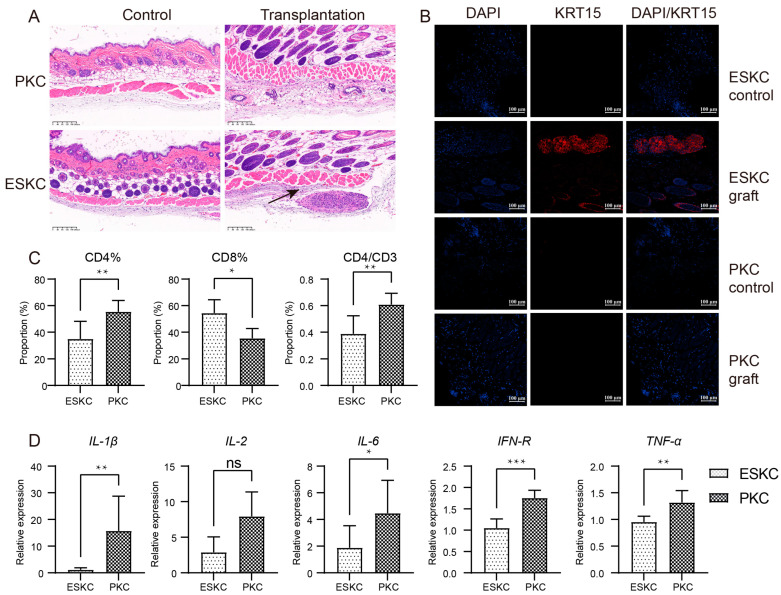
In vivo comparison of immunogenicity between ESKCs and PKCs. (**A**) Microscopic images of HE staining on skin sections from the transplantation site. The control group refers to the subcutaneous tissue on the non-transplanted side, while the transplantation group refers to the subcutaneous tissue on the transplanted side. The black arrows indicate surviving cell clusters of ESKCs. (**B**) The comparison of key keratinocyte-related markers in skin graft sections at the transplantation site under confocal microscopy. (**C**) The comparison of the immune profile of the spleens in different transplantation groups. (**D**) The comparison of mRNA transcription of critical inflammatory cytokines in immune cells of the spleens in different transplantation groups. The relative expression levels representing the mRNA relative expression levels of each group relative to the reference gene TBP within the ESKC transplantation group. Numeric comparisons between groups were conducted using *t*-tests. Data in the figures are presented as mean ± S.D of n = 3; *, *p* < 0.05; **, *p* < 0.01; ***, *p* < 0.001; ns, no statistical significance.

One week after transplantation, the skin of humanized immune model mice transplanted with ESKCs displayed a clear structure with distinct layers, and no apparent inflammatory response was observed. Subcutaneous cell clusters were present beneath the smooth muscle layer in the skin within the ESKC transplantation area in HE staining. KRT15 is one of the specific markers for keratinocytes, and its expression is low in the smooth muscle layer and deeper regions of the normal mouse skin [22]. Immunofluorescence staining showed that these cell clusters exhibited a significant expression of KRT15. In contrast, similar cell clusters were not found in the skin tissue in the PKC transplantation group. Immunofluorescence staining showed that the PKC transplantation group did not reveal the presence of KRT15-expressing cell clusters (Figure 6A,B). These results indicated that ESKCs can survive subcutaneous transplantation into humanized mice for one week, suggesting that ESKCs demonstrated a relatively low immunogenicity.

Murine spleen cells were extracted and subjected to flow cytometry analysis. The results indicated that the ESKC group had a significantly lower proportion of CD4^+^ T lymphocytes among total spleen cells, and there was also a lower proportion of CD4^+^ T lymphocytes among total CD3^+^ T lymphocytes (Figure 6C). The immune cells in the ESKC transplantation group exhibited significantly lower gene expression levels of IL-1β, IL-6, IFN-γ, and TNF-α (Figure 6D).

## 4. Discussion

Clarifying the immunogenicity of differentiated cells from embryonic stem cells is crucial for their future use in transplantation. In this study, we successfully differentiated keratinocytes from hESCs and demonstrated that they exhibit lower immunogenicity compared to primary keratinocytes.

Immune responses are triggered by the interaction between immune molecules and ligands. Our data indicated that ESKCs exhibited a significantly lower expression level of immune-related molecules. HLA is involved in the induction of allogeneic tissue or organ transplantation rejection. When T cells recognize HLA molecules on donor cells with their TCRs, they will be activated and trigger immune responses [23]. In this study, we found that the expression of HLA class I molecules in ESKCs was lower than that in PKCs. And the expression of genes associated with HLA-I molecules such as MFSD6 and MR1 was also lower in ESKCs than in PKCs. MFSD6 predicts and enables HLA-I protein binding activity and HLA-I receptor activity involved in the processing and presentation of exogenous peptide antigen via HLA-I [24]. MR1 was related to antigen binding and HLA-I receptor activity [25]. We speculated that the low expression of these genes was a relevant factor for the low expression of HLA-I molecules in ESKCs. We also observed that the HLA-I expression in ESKCs can be upregulated to levels similar to those in PKCs when stimulated by IFN-γ, but not when stimulated by TNF-α. The mechanism responsible for this difference is unknown.

As keratinocytes are not professional antigen-presenting cells [26], both ESKCs and PKCs exhibited low basal expression levels of HLA-II molecules. Under IFN-γ stimulation, PKCs showed a remarkably high level of HLA-II molecule expression, consistent with the characteristic of keratinocytes as non-professional antigen-presenting cells. But ESKCs were different, as ESKCs did not exhibit a significant upregulation of HLA-II molecules under equivalent conditions of IFN-γ stimulation. The reduction in HLA-II molecule expression will impact the activation and proliferation of CD4+ T lymphocytes, indicating a weaker capacity to initiate alloimmune responses [27]. This result suggests that, unlike PKCs, ESKCs may have low immunogenicity to external stimulation.

It is reported that the molecular mechanism underlying IFN-γ-stimulated HLA-II molecule expression may involve the activation of the STAT1/IRF1 pathway [28]. The STAT1 dimer is transported into the cell nucleus through IFN-γ-NPI-1 transport proteins, where it binds to IFN-γ activation sequence (GAS) sites, thus activating the transcription of IRF. IRF1 and IRF2 cooperatively activate CIITA, allowing CIITA to work in conjunction with various other transcription factors to collectively promote the transcription and elongation of HLA-II genes, resulting in enhanced HLA-II molecule expression [29,30]. Accordingly, the present study discovered significantly lower expression of STAT1, IRF1, and IRF5 genes in ESKCs. These results may explain the differences in the expression of HLA molecules between ESKCs and PKCs when the cells were stimulated by IFN-γ.

Additionally, co-stimulatory molecules are crucial factors in immune responses. These molecules influence T lymphocyte proliferation and function by binding to corresponding receptors on the surface of T lymphocytes [31]. Our results showed that ESKCs maintained low expression levels of CD40, CD80, and CD86 molecules even under inflammatory cytokine stimulation, underscoring the weak antigen-presenting capacity of ESKCs.

Previous research has indicated that during acute allograft rejection in human organ transplantation, CD4+ lymphocytes in peripheral blood significantly increased, while the number of CD8+ lymphocytes decreased [32]. In the present study, we have observed that ESKCs have a lower capacity to stimulate the proliferation of T lymphocytes, particularly CD4+ T lymphocytes whether in vitro or in vivo. This phenomenon may be associated with the low expression of HLA-II molecules in ESKCs. The expression and secretion levels of cytokines also serve as indicators of lymphocyte activation and the immune response in transplantation. ESKCs exhibited significantly lower ability to stimulate pro-inflammatory cytokines secretion such as IL-1β, IL-6, IFN-γ, and TNF-α, compared to PKCs. Moreover, ESKCs induced higher expression levels of the anti-inflammatory cytokine IL-10 secretion. These results collectively indicated that ESKCs have lower immunogenicity compared to PKCs. Although humanized immune model mice can, to some extent, represent the human immune system for testing allograft rejection reactions, it is essential to recognize that there exists a certain disparity in the composition of immune cells between animal models and the human immune system [33]. Additionally, animal model observations are subject to a specific window period, and currently, there is a limitation in conducting prolonged observations of chronic rejection reactions [34]. Furthermore, the study by Drukker et al. indicated that the H9 and H13 human embryonic stem cell lines express HLA class I molecules, while the expression of HLA class II molecules is minimal [35]. Grinnemo et al. investigated the HLA molecule expression levels in various hESC lines, such as HS237 and HS293, and obtained similar results [36]. In this study, the HLA molecule expression in the H9 cell line is consistent with previous research findings. However, whether the keratinocytes derived from other hESC lines also exhibit similar immunogenic characteristics remains to be further explored. Therefore, further research and validation are still required to understand the immune responses of ESKCs in in vivo transplantation.

## 5. Conclusions

This study proved that the keratinocytes differentiated from human embryonic stem cells exhibited low immunogenicity in alloimmune reactions, suggesting their potential as transplantation cells in the field of regenerative medicine.

## Figures and Tables

**Table 1:** Mixed lymphocytes reaction.

	Group	Stimulating Cells	Responder Cells
		1 × 10^5^ Cells per Well	1 × 10^6^ Cells per Well
1	hESCs	hESCs	PBMCs-A
2	ESKCs	ESKCs	PBMCs-A
3	PKCs	PKCs	PBMCs-A
4	NC	PBMCs-A	PBMCs-A
5	PC	PBMCs-B	PBMCs-A

(1) hESCs: Human embryonic stem cells; (2) ESKCs: Keratinocytes derived from human embryonic stem cells; (3) PKCs: Primary human keratinocytes; (4) NC: Negative control; PBMCs-A: PBMCs from healthy volunteer A; (5) PC: Positive control; PBMCs-B: PBMCs from healthy volunteer B.

## Data Availability

The original contributions presented in the study are included in the article; further inquiries can be directed to the corresponding authors.

## Supplementary Materials

The following supporting information can be downloaded at the following: https://www.mdpi.com/article/10.3390/cells13171447/s1, Figure S1: Gating strategy for flowcytometry; Figure S2: (A) Peripheral blood immune profiling of human PBMC reconstituted in NOG-dKO mice. (B) Comparison of peripheral blood immune profiles in NOG-dKO mice after randomization into two groups; Figure S3: The fluorescence intensity of HLA molecules in ESKCs, PKCs and MoDCs. Table S1: Experimental materials; Table S2: Primers for qPCR.
