# Supplementary material for: Low Immunogenicity of Keratinocytes Derived from Human Embryonic Stem Cells
Source: Cells. 2024 Aug 29;13(17):1447. doi: 10.3390/cells13171447 (PMC11393835; doi:10.3390/cells13171447)
Supplement: Supplementary file 1 [file cells-13-01447-s001.zip › cells-3076214-supplementary.pdf]

Supplementary Table S1 Experimental materials

name	Cat./No.	Supplier
BMP-4	CYT-081	Prospec
BMP-7	CYT-333	Prospec
Collagen I	C4243	Sigma
Dexamethasone	D4902-25MG	Sigma
DMEM/F12	L310KJ	BasalMedia
DNeasy Plant mini kit	69104	Qiagen
Donkey Anti-Mouse IgG H&L (Alexa Fluor® 647)	ab150107	Abcam
eBioscience™ Intracellular Fixation & Permeabilization Buffer Set	88-8824-00	Invitrogen
IGF -1	P5502	Beyotime
ITGA6	ab181551	Abcam
ITS-X	S452	BasalMedia
KRT14	ab119695	Abcam
KRT6C	ab227643	Abcam
NAOH	A620617	Sangon
PBS	B210KJ	BasalMedia
PrimeScript™ RT Master Mix	RR036A	TAKARA
RA	R2625	Sigma
RNeasy mini kit	74104	Qiagen
TB Green Premix Ex Taq	RR420A	TAKARA
Trizol	15596026	Thermo Fisher
Matrigel	354277	CORNING
2.0 mL EP tube	mct-200-c	Axygen
15 mL centrifuge tube	601051	NEST
50 mL centrifuge tube	602052	NEST
96-well plate	4346907	Thermo Fisher
8-channel pipette	A30588	Thermo Fisher
Confocal dish	801002	NEST
Flow cytometry tube	352235	CORNING
6-well plate	3516	CORNING
Centrifuge	Centrifuge 5420	eppendorf

Confocal microscope	LSM800	Zeiss
CO2 incubator	Forma	Thermo Fisher
Micropipette	Research® plus	eppendorf
Flow cytometer	LSRFortessa™ Flow Cytometer	BD bioscience
Centrifuge	Centrifuge 5427 R	eppendorf
Q-PCR machine	QuantStudio™ 3 Real-Time PCR System	Applied Biosystems
Gene sequencer	Hiseq 6000	Illumina
Gene sequencer	RSII	Pacbio
Nucleic acid concentration detector	Colibri LB 915	Berthold Technologies
CO2 incubator	Forma 3111	Thermo Fisher
Microplate washer	Scientific Wellwash Versa	Thermo
Fluorescence scanner	InnoScan 300 Microarray Scanner	Innopsys
PE anti-human ITGA6	ab308050	Abcam
APC anti-human KRT14	ab7800	Abcam
FITC anti-human KRT5	ab64081	Abcam
APC anti-human CD3	317317	Biolegend
FITC anti-human CD8a	300905	Biolegend
PE anti-human CD4	980804	Biolegend
PE anti-human CD11c	980602	Biolegend
APC anti-human HLADPDRDQ	980406	Biolegend
FITC anti-human HLA-ABC	311403	Biolegend
FITC anti-human CD40	334305	Biolegend
FITC anti-human CD80	375405	Biolegend
FITC anti-human CD86	374203	Biolegend

Supplementary Table S2 primers for qPCR

Gene	primer（5’-3’）
GAPDH-F	GGAGCGAGATCCCTCCAAAAT

GAPDH-R	GGCTGTTGTCATACTTCTCATGG
IRF1-F	ATGCCCATCACTCGGATGC
IRF1-R	CCCTGCTTTGTATCGGCCTG
IRF5-F	GGGCTTCAATGGGTCAACG
IRF5-R	GCCTTCGGTGTATTTCCCTG
IFIH1-F	TCGAATGGGTATTCCACAGACG
IFIH1-R	GTGGCGACTGTCCTCTGAA
MR1-F	GATGGCGTTCCTGTTACCTCT
MR1-R	GCCCAGGCGAAAATATCTCAG
MFSD6-F	TCCAAGCCAGAGTGGACTACT
MFSD6-R	GGTTTGACAAATCCAATGCCC
TBP F	CCACTCACAGACTCTCACAAC
TBP R	CTGCGGTACAATCCCAGAACT
IL-1β F	ATGATGGCTTATTACAGTGGCAA
IL-1β R	GTCGGAGATTCGTAGCTGGA
IL-2 F	CTCACCAGGATGCTCACATTT
IL-2 R	ACTTCCTCCAGAGGTTTGAGT
IL-6 F	ACTCACCTCTTCAGAACGAATTG
IL-6 R	CCATCTTTGGAAGGTTCAGGTTG
IFN-γ F	TCGGTAACTGACTTGAATGTCCA
IFN-γ R	TCGCTTCCCTGTTTTAGCTGC
TNF-α F	GAGGCCAAGCCCTGGTATG
TNF-α R	CGGGCCGATTGATCTCAGC

Supplementary Figure S1 Gating strategy for flowcytometry

A

	Antigen	cat.	supplier
Keratinocytes	PE anti-human ITGA6	ab308050	abcam
	APC anti-human KRT14	ab7800	abcam
	FITC anti-human KRT5	ab64081	abcam

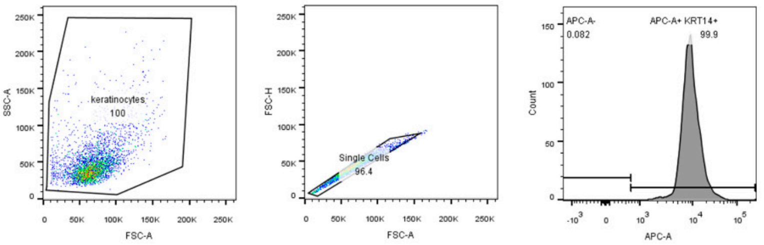

B

	Antigen	cat.	supplier
stimulated keratinocytes	APC anti-human HLA-DPDRDQ	980406	biolegend
	FITC anti-human HLA-ABC	311403	biolegend
	FITC anti-human CD40	334305	biolegend
	FITC anti-human CD80	375405	biolegend
	FITC anti-human CD86	374203	biolegend

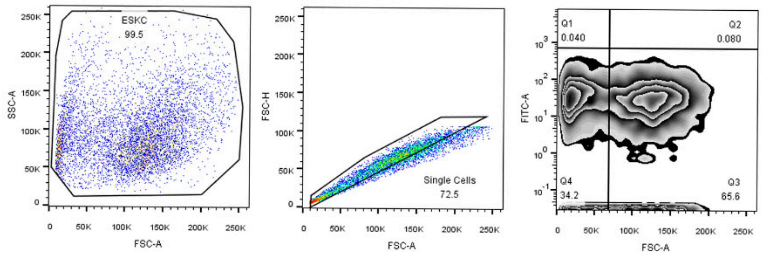

C

	Antigen	cat.	supplier
T cell	APC anti-human CD3	317317	biolegend
	FITC anti-human CD8a	300905	biolegend
	PE anti-human CD4	980804	biolegend
DC	PE anti-human CD11c	980602	biolegend
	APC anti-human HLADPDRDQ	980406	biolegend

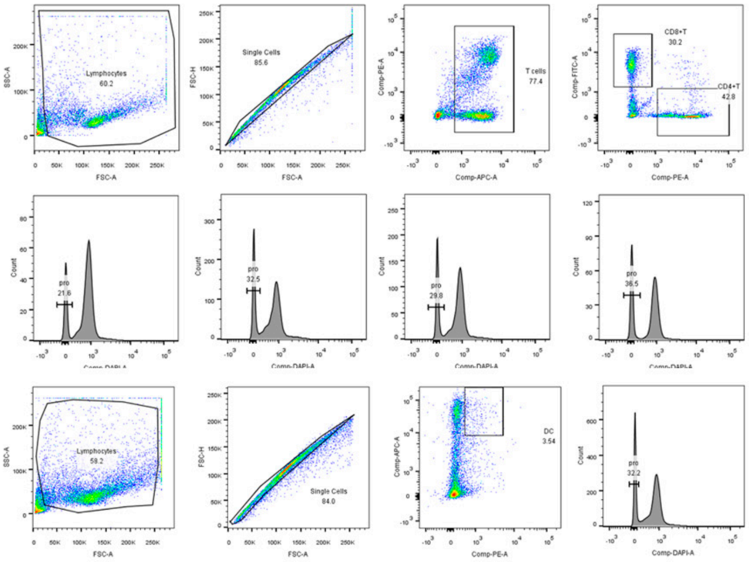

Figure S1 Gating strategy for flowcytometry

(A) Gating strategy of keratinocyte-related markers

(B) Gating strategy of HLA and co-stimulatory molecules

(C) Gating strategy of T cells and DCs

Supplementary Figure S2

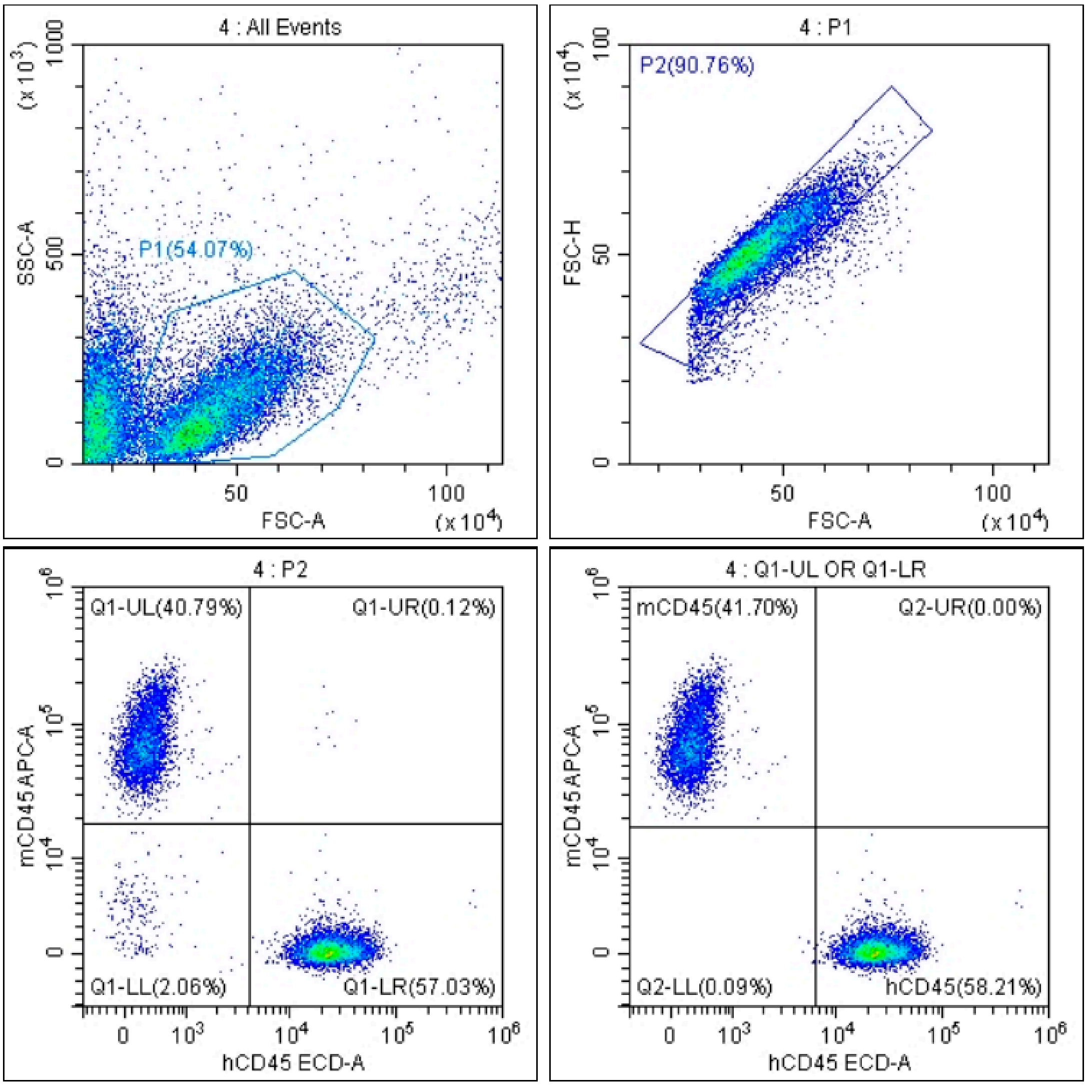

Figure S2A Peripheral blood immune profiling of human PBMC reconstituted in NOG-dKO mice

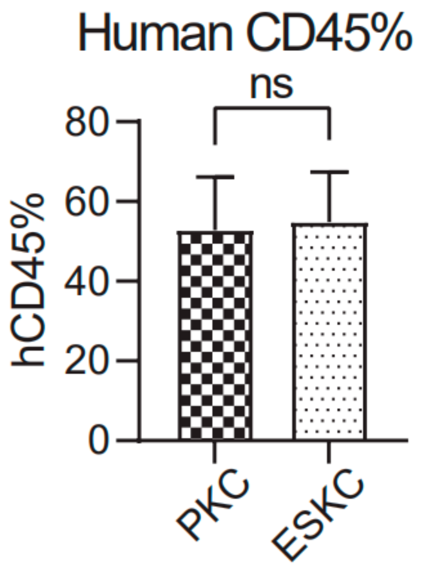

Figure S2B Comparison of peripheral blood immune profiles in NOG-dKO mice after randomization into two groups

Supplementary Figure S3

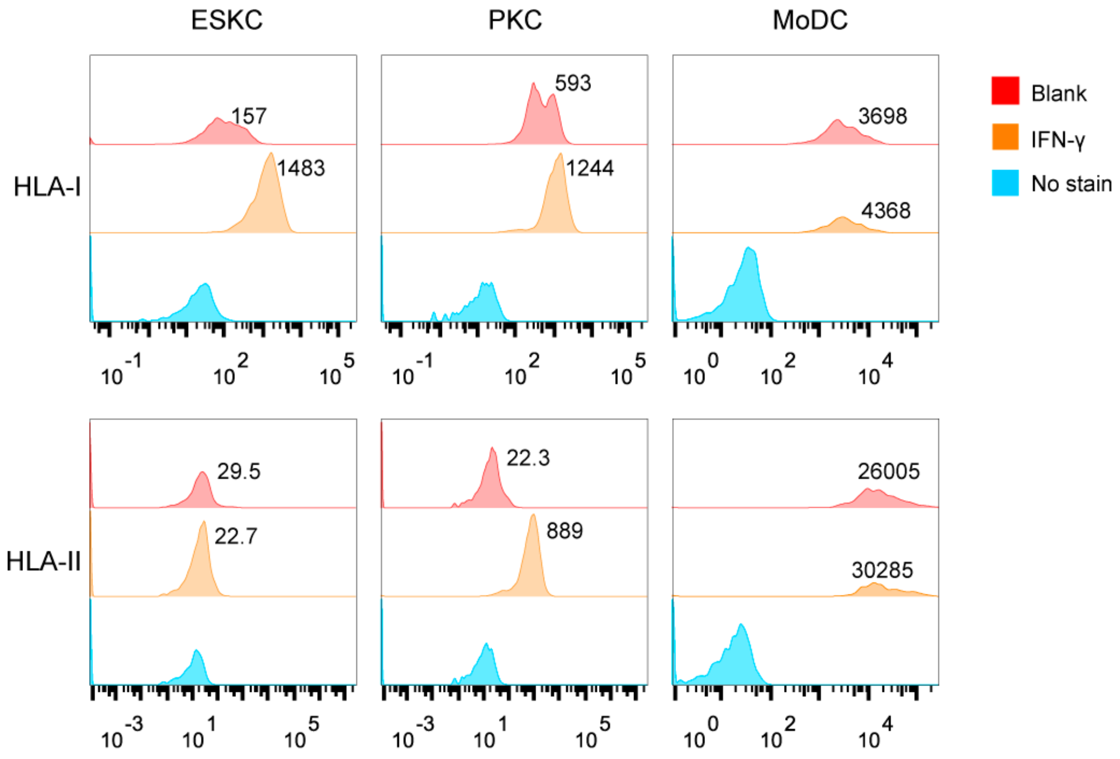

Figure S3 The fluorescence intensity of HLA molecules in ESKCs, PKCs and MoDCs. The mean fluorescence intensity for each sample is indicated next to the peak of the histogram.
